# Burn Care in the Greek and Roman Antiquity

**DOI:** 10.3390/medicina56120657

**Published:** 2020-11-28

**Authors:** Christoph Wallner, Eric Moormann, Patricia Lulof, Marius Drysch, Marcus Lehnhardt, Björn Behr

**Affiliations:** 1 Department of Plastic Surgery, BG University Hospital Bergmannsheil, Ruhr University Bochum, Bürkle-de-la-Camp Platz 1, 44789 Bochum, Germany; marius.drysch@rub.de (M.D.); marcus.lehnhardt@rub.de (M.L.); bjorn.behr@rub.de (B.B.); 2 Department of Archaeology, Amsterdam Centre of Ancient Studies and Archaeology-ACASA, University of Amsterdam, Turfdraagsterpad 9, 1012 XT Amsterdam, The Netherlands; P.S.Lulof@uva.nl; 3 Department of History, Art History and Classics, Radboud University, Erasmusplein 1, 6525 HT Nijmegen, The Netherlands; e.moormann@let.ru.nl

**Keywords:** burn, care, antiquity, phytotherapy, ancient medicine

## Abstract

The last century brought about more rapid new developments in the treatment of burns, which significantly lowered the mortality of burn injuries. However, burns were already treated in antiquity, where the threshold from spirituality to scientific medicine originated. The existing literature on burn treatment is very limited and there are many cross-references, some of them incorrect. The aim of this work by an interdisciplinary team of historians and physicians is to offer a more precise reproduction of the burn treatment of Greek and Roman antiquity using original texts in context and with a modern scientific background. There are many sources from ancient doctors on the subject of burn treatment, as well as the treatment of burned-out wounds and frostbite, which have not yet been mentioned. The literature research also showed an understanding of scientific contexts in ancient medicine, such as antiseptics or rheology. Interestingly, there was a change in burn medicine from everyday Greek medicine to Roman military medicine with other burn patterns. The care of patients using analgetics and the therapy of burn shock arose from the literature. The ancient world is considered to be the foundation of medicine, but it is believed to have been based mainly on shamanism rather than science. However, already more than two millennia ago, burns were correctly assessed and treated according to today’s scientific standards and scientific relationships were recognized.

## 1. Introduction

Over the past centuries but especially in recent decades, the advances in burn care have led to a massive improvement of clinical care and therefore to a decrease in mortality of burn patients [1]. While innovations in modern medicine reduce the mortality of those with severe burns to less than 5%, new challenges arise from, for example, multi-drug resistant microorganisms. Burn patients with large wound areas and relatively long mechanical ventilation are particularly susceptible to these germs and exposed to them without protection. While modern concepts fail, very old methods of treating burn injuries are used, which, like medical honey, overcome multi-drug resistant microorganisms [2,3]. The use of medicinal honey in the treatment of wounds was first recorded in ancient times. On the one hand, through the writing of medical treatments but also through the technological progress of the time—there was a medical revolution in antiquity. In this article, we want to focus on the treatment of burns in the Greek and Roman antiquity. There is little translated literature available from Greek and Roman antiquity about the treatment of burns. We therefore see a great opportunity in summarizing the existing medical literature as well as in translating unnoticed texts on the therapy of burn injuries from this time.

To include a relevant body of modern medical literature, a search on pubmed with the MeSH ““Burns/history” [MAJR]” was performed on 26 September 2020 including 144 results, of which 12 results included the era of the Greek and Roman antiquity. For the classical literature review, texts of Hippocrates of Kos, Galen of Pergamon, the Alexandrian school (Heracleides of Tarentum, Sostratos, Herophilos of Chalkedon, Praxagoras of Kos and others), Pedanios Dioscurides, and Aulus Cornelius Celsus were considered. In addition, “Der Kleine Pauly” as the standard reference work of antiquity served as the basis for the extended search.

### 1.1. Animal Products

On pubmed, only a small number of references to the medical treatment of burns in antiquity and an even smaller number referring to Roman and Greek antiquity was found. Interestingly, animal products have been a main source for the local treatment of burns. In early antiquity, Egyptians used goats milk and milk from women who had given birth to a son [1]. It has been shown in recent literature that milk not only has antibacterial and antiviral properties but also pro-regenerative capacity [2,3,4]. This might have been the observation in early antiquity leading to the long tradition in Roman antiquity, middle ages and modern times of using milk and derived products like butter or oil for the treatment of wounds in general. According to modern knowledge, fresh milk in particular has a bactericidal and antiseptic effect due to the milk compounds lactoferrin and β-casein [2,5,6,7].

The earliest account of using parts of a dead animal is recorded in the Papyrus of Ebers 1500 BC, by using a warmed frog in oil and rubbed onto a burned surface [8]. Around 430 BC, Hippocrates reported using pig lard as a treatment option for burns and referred to a better appearance of scars [9] (p. 367 et sqq.):

*To soften you must employ the following medications–more so in winter than in summer–medications for softening, which also make the scars neat. Pound this internal pulpy material of the squill, or pine bark with fresh lard, squill and a little olive oil. Whitest wax and fresh clean grease. Or squill white olive oil, verdigris, squill and resin: let there be two parts of the aged lard and of the other components as much as seems appropriate. Melt fresh grease, pour it off into another cup, grind galena very fine, sift, mix the two together and boil stirring at first; boil until when dripped on to the shavings of nettle-tree wood and red ochre: anoint with this, then boil leaves of cuckoo-pint in wine and olive oil, appl them to the burn and bind them in place with a bandage.*

**Figure 1:**
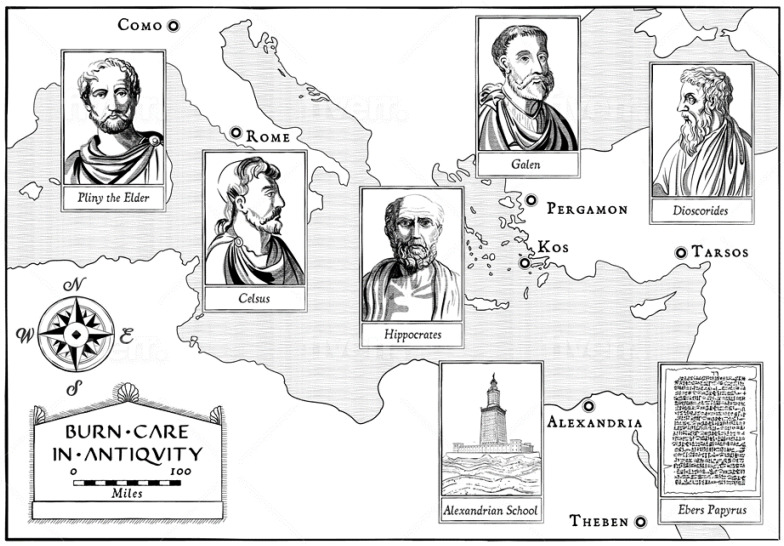
Origin of the various sources for the treatment of burns in ancient times: The Ebers Papyrus is one of the earliest medical records ever (1500 BC) and already contains the treatment of burns. While Hippocrates was active in ancient Greece around 400 BC, in Hellenism, the scientific elite gathered in the Alexandrian School at the mouth of the Nile. In the early Roman Empire, Pliny the Elder, Disorides, and Celsus had a major impact on medical progress. Around 150 AD, Galen led another surge in medical research. All of them contributed substantially to the treatment of burns.

Already in Egyptian literature, the treatment of burns with honey and grease was documented on the Papyrus Edwin Smith around 1500 BC [10] (see Figure 1). In ancient Greece, honey (known as “nectar of Gods”) was also mixed with vinegar, alum, sodium, carbonate and bile. This was known as *enheme*—to desiccate a wound and prevent suppuration [11]. Celsus, around 30 AD, proposed honey combined with bran as a topical remedy for burn wounds. The *Materia Medica* by Pedanios Dioscurides, a Greek physician in the Roman army, consists of five books, and is considered the most important and influential ancient work on medicines (origin see Figure 1). Dioscorides designated honey as the treatment of choice for the treatment of rotten and hollow ulcers [12] (bk.1). He recommended to change the dressings on a frequent basis. Interestingly, indigenous people of Australia and the Americas also used honey as a treatment for wounds. Today, it is scientifically acknowledged that honey, with its antibacterial properties—mostly through its glucose oxidase, methylglyoxal and bee defensin-1, anti-inflammatory, antioxidant and pro-regenerative capacity, reduces infections on burns and accelerates the healing [13,14].

Dioscorides wrote about using a conch shell and burned leather from shoes as treatments for burn wounds [12] (bk.2; paragraph 8/51):

*The roasted trumpet snails, they are even more caustic. If you burn them in a raw pot filled with salt, they make a good means for brushing your teeth and as a poultice for burns. But you have to let the remedy burn as hard as a broken glass; after the burn wound has scarred, it falls off by itself.*

*The leather of old shoe soles, burned and finely pounded, heals as Envelope burns, decubitus and the pressure of shoes caused inflammation.*

He also wrote down various recipes of cattle droppings for the treatment of purulent burns. This has been used in conjunction with rose oil and honey for inflamed burn wounds [12] (bk.2; paragraph 98). At this point, it must be mentioned that many substances have been tried that, from today’s perspective, have a negative effect on wound treatment; however, in combination with antiseptic substances such as rose oil or honey, these ingredients were wrongly identified as good wound topicals. Interestingly, cow dung was identified to be antiseptic through its methanol, hydro alcohol and aqueous compounds [15].

### 1.2. Plant-Based Products

Around the year 3000 BC, an unknown Sumerian physician documented several plant-based remedies. The Papyrus of Ebers described, around 1500 BC, the topical application of lemon stripes on burn wounds. A new insight revitalizes the findings of Hippocrates that suggest vinegar is an effective treatment of burns to avoid infection. Acetic acid is capable of treating particularly pseudomonal wound infections and can break down biofilms [16,17]. Hippocrates was also known to use red wine with similar effects to treat burn wounds [9] (p. 367 et sqq.). In the first century AD, Celsus described the use of wine and myrrh as a burn lotion, which is known to have bacteriostatic properties [18]. Celsus also commented on inflammation in his book *De medicina* [19] (bk.5; Chapter 2):

*The following subdue inflammation: alum, both split alum called schiston, and alum brine; quince oil, orpiment, verdigris, copper ore, blacking.*

Today, myrrh is scientifically proven to treat complicated wounds and ulcers, especially in very moist wounds. It is similarly effective against most Gram-negative strains, Gram-positive strains and even yeast compared to modern antibiotics [20]. In 430 BC, Hippocrates used bitumen mixed with resin to treat advanced cases of burns. It has been shown that resin of certain plants leads to increased angiogenesis in an animal burn study [21]. Resin of most plants is antibacterial as it is well known to interfere with reproduction of parasitic microbes. *E. faecalis* was most sensitive and *S. typhi* was resistant to *myrrh.* The extracts of *O. turpethum* were active against all tested strains in which *B. subtilis* and *S. aureus* were the most sensitive [22].

With the Roman expansion, the treatment of war injuries attracted the interest of many physician. Gaius Plinius Secundus recommends using garlic (raw or cooked) onto burn wounds, as the alliin contained in it, later discovered, has an antibiotic effect (Plinius, XX, 51: Suspiriosis coctum, aliqui crudum id dedere).

Dioscorides alluded that oil from the immature *Olea europaea* called *Omphakion* can be used as an ointment and heals wounds. Recent studies showed increased wound contraction and wound tensile strength when treated with extracts of *Olea europaea* [23]. In modern day studies, olive oil exhibited substantial wound healing efficacy in chronic wounds [24]. However, the antimicrobial capacity is limited [25]. Dioscorides also reported using incense *Boswellia sacra* or *Asphodelus racemosusin* in multiple ways and was one of the first to mention frostbites [12] (bk.1; paragraph 81) [12] (bk.2; paragraph 199):

*It has the power to warm up, to astringent, to drive away the darkenings on the pupils, to fill in the hollow areas of the wounds and to scar them, to glue up bloody wounds, to hold back all blood flow, including that from the brain. Rubbed and spread on charpie with milk, it soothes the malignant ulcers around the anus and the other parts; In the beginning, spreading it with vinegar and pitch, it drives away the warts and lichen. With pork or goose lard he also heals burned-out ulcers and frost damage. He cures bad grind together with nitrum (soda), paronychia with honey, bruised ears spread with pitch, against the other ear complaints he helps with sweet wine. It heals inflammation of the breasts from birth as an ointment with cimolic earth and rose oil.*

*If oil (Asphodelus racemosusin) is heated in a fire in the hollowed-out roots, it helps with burns and ulcerated chilblains…*

After the burnout of wounds developed its long tradition in the Roman army, the treatment of burned-out wounds was described in the texts accordingly. Dioscorides wrote about treating burned-out wounds with bark from the pine [12] (bk.1; paragraph 86), which contains antiseptic alkaloids, as modern research has shown [26,27]:

*The pine is a well-known tree; that belongs to the same species spruce, which is different in appearance from it.*

*The bark of both is contracting; when sprinkled on top of it, it is a good remedy for Wolf, and likewise with black lead and manna for granulation growths and burned-out wounds.*

However, he also writes clearly about burns and their treatment with grinded blossoms of the *Cistus creticus* and pickled olives to avoid blistering with [12] (bk.1; paragraph 126/138):

*For themselves as an envelope, they hold up eating ulcers. With wax ointment, they heal burns and old wounds.*

*Canned, finely pounded olives are used as a poultice for burns with fire, preventing blistering and cleaning dirty wounds.*

He also differentiated superficial and deep burn wounds and suggests to put fig together barley flour onto sun burns [12] (bk.1; paragraph 183). Barley flour might have antiseptic and wound healing properties due to a high level of antioxidants [28].

### 1.3. Chemical Products

Without knowing about germ theory, silver was used in ancient folklore medicine. Silver was used for water storage and water disinfection [29]. In 350 BC, Alexander the Great only drank water from silver vessels. When a more precise definition of the wounds was lacking, silver nitrate was used by Roman physicians as a remedy [29,30]. It has not yet been proven whether this was also used for burns. Silver is shown to be highly effective against Pseudomonas, Streptococcus and Staphylococcus [30]. Due to the introduction of silver sulfadiazine in the 1960s, silver was the standard topical antimicrobial for burn wounds for decades. In the past decade, however, silver sulfadiazine has been superseded by other more effective agents [31,32].

Dioscorides mixed sulfur together with Carthamus corymbosus to avoid spots of sunburns [12] (bk.3; paragraph 9). He also described how the Phyrigian stone was applied to the wound to remove eschar and was combined with wax ointment to heal burn wounds [12] (bk.5; paragraph 140).

### 1.4. Aspects of Patient Care

Hippocrates mentioned the importance of aseptic conditions when treating burn wounds as well as the proper supply of fluids per os diluted with honey. He also described septicemia after burn injury [9] (p. 367 et sqq.):

*In this condition acute fever and increased pulse rate occurred” … “In major burns, spasm or tetanus are poor prognostic signs” … “Rigors with delirium lead to death.”*

For analgesia and wound cleaning, Hippocrates applied sea water to the wound. It is also described that he used red wine to clean the wound. As described before, Hippocrates and Galen used vinegar for antiseptic burn treatment, but its evaporation and cooling effect was an effective local analectic [1]. Celsus documented the use of various analgesic plants, including poppy seeds with the alkaloids morphine, codeine, and papaverine they contain. Overdose was described by Gaius Plinius Secundus and Dioscorides [33] (bk.20; p. 198 et sqq.). Analgesics for operations performed at the time may also have been used to treat burns. These include hemp, deadly nightshade and mandrake [12] (bk.4; 69 et sqq.). In modern anesthesia and consequently burn medicine, opioids have become as indispensable as strong analgesics. Synthetic derivatives of morphine, such as sufentanil, are used in everyday operations and anesthesiology. In 2018, 388.2 tons of morphine was produced and either consumed or processed into derivatives; 1.5 tons of fentanyl, which is about 100 times stronger than morphine, was consumed in 2018 [34] (p. 30).

In his fifth book *De Medicina,* Celsus explained the differentiation and properties of different debris associated with inflammation and the application of vinegar [19] (bk.5, Chapter 26). He also mentioned the surgical excision of contracted scars in burn wounds [19] ( bk.5, Chapter 26).

Dioscorides had also referred to pediatric burn injuries and developed recipes specifically for children [12] (bk.4; paragraph 71):

*(Solanum nigrum)…spread with rose ointment, it is a good remedy for children who suffer from sunburn.*

Celsus provided the first description of the four cardinal signs of inflammation “rubor”, “calor”, “tumor” and “dolor”, which are applicable for burn wounds too. Galen of Pergamon added the “functio laesa” around 150 AD.

### 1.5. Summa Summarum

Through our review of modern literature on ancient Mediterranean medicine, we found a lot of cross-referencing with a small number of reliable translations. Some descriptions only mention a spiritual or alternative medical therapy of burns in this time period [35]. The translation of ancient texts could shed new light on the treatment of burns in ancient times with a huge variety of strategies to treat burn wounds, to control infections but also to treat the patient during a burn-associated shock. Ancient medicine has to be viewed in the context of non-written, passed down and thus actually non-existent medicine, before the transition of medicine into the Middle Ages happened with the accompanying loss of established and successful therapies. Many of them, i.e., the medical honey, silver and plant extracts, have been reintroduced into modern evidence-based medicine in the past 100 years.

We assume that ancient Greek medicine developed based on observation and so, for the first time in history, it represents evidence-based medicine. There seem to be many references to local therapeutics with little religious reference, whereas in ancient Egypt, gods were mostly invoked and therapeutics were used generically.

However, when translating Dioscorides Materia medica, it can be seen that some recommended recipes (using ingredients that are today identified as harmful like livestock manure) were only able to work together with other antiseptic ingredients, for example, rose oil.

Interestingly, the clinical pictures and applications vary from the Greek sources described as everyday injuries to the military medicine described in the Roman sources. This is elucidated, for example, through the burning out of gunshot wounds and their treatment as well as frostbites, which were described in the Caesarean Germanic or British wars. Thousands of years later, the First and Second World Wars in particular, significantly revolutionized wound treatment through innovations such as penicillin, modern surgery and anesthesia.

### 1.6. Legacy of the Ancient World

In the centuries that followed, it was not only a lack of development in the treatment of burns that happened but also a retrograde doctrine took place assuming that suppuration was essential and helpful to healing, as proposed by Galen. Avicenna also propagated the pre-Hippocratic aphorism that diseases that cannot be cured by iron are cured by fire. It took as long as until the late Middle Ages until medical professionals as barbers reintroduced the principles of Hippocrates’ “antiseptic” treatment of burns [36]. Despite modern medical research, burn medicine is facing new challenges such as multi-drug resistant microbes and, in view of the increasing comorbidities, wounds that are difficult to treat. Today’s burn medicine is rediscovering ancient recipes to face these challenges.

## 2. Conclusions

In summary, ancient medicine gave birth to scientific thinking and acted as the beginning of evidence-based medicine. There was a lively exchange and correspondence between the different schools. The meetings between active physicians in centers (e.g., Alexandria) as well as the adoption of concepts over generations in the whole Mediterranean area is documented [37]. Burn medicine stands, pars pro toto, for the development, testing and application of this newly emerging thinking. Unlike before, the favor of the gods was no longer relied on in curing various diseases, but observations of nature were implemented and further developed in the context of critical assessment for a successful medical treatment.
